# 

**Published:** 1858-12

**Authors:** 


**RECEIPTS FOR JOURNAL.**

Jno. R. Eypert, Tenn., $3 ; R. S. Payne, Va., $9 ; S. C. Farrar, Miss., $9 ; J. M. McMichael, Ala., $5 ; R. A. Fontaine, Ga., $2 ; Brooks & Jones, Ark., $3 ; P. S. Carpenter, Ala., $9 ; J. M. Heard, Miss., $3 50 ; E. L. Burton, Ga., $12 ; A. Ellis, Ga., $3 ; M. B. Bogan, Ga., $6 ; Jno. B. Johnson, Mo., $6 ; F. H. Evans, Miss., $6 ; W. Somerville, Va., $6 ; M. O. Stribling, Ga., $3 ; S. H. Freeman, Ga., $3 ; D. F. Dickinson, Ga., $8 ; W. F. Shelton, Ga., $9 ; S. D. Wheat, Ga., $3 ; R. B. Jackson, Ala., $6 ; N. H. Baker, Ala., $3 ; Cowden & Hughes, Ala., $9 ; H. R. Casey, Ga., $9 ; M. L. Pool, Ga., $2 ; Martyn Paine, N. Y., $3 ; J. M. Couch, Ga., $5 ; B. M. Thomson, Ga., $2 ; L. P. Garrison, Ga., $2 ; John Gay, Ga., $9 25 ; Kendrick & Nesbit, Ala., $9 ; Thomason & Wilcoxon, $9 ; Jos. E. Harvey, Miss., $3.

